# Technical note: sensitivity analysis of the SCoRE and SARA methods for determining rotational axes during tibiofemoral movements using optical motion capture

**DOI:** 10.1186/s40634-020-0219-z

**Published:** 2020-02-10

**Authors:** Michèle N. J. Keizer, Egbert Otten

**Affiliations:** University of Groningen, University Medical Center Groningen, Center of Human Movement Sciences, Antonius deusinglaan, Groningen, 9713 AV The Netherlands

**Keywords:** Functional axis of rotation, Knee laxity, Tibia translation, Tibia rotation

## Abstract

**Purpose:**

The first aim was to report the sensitivity of calculated tibiofemoral movements for the choice of placement of the set of femoral markers. The second aim was to report the influence of accuracy of the motion captured positions of the markers on the calculated tibiofemoral movements.

**Methods:**

Tibiofemoral kinematics during single leg hops for distance were calculated. For the first aim, an experiment was conducted in which four different setups of the femoral markers were used to calculated tibiofemoral movements. For the second aim, an experiment was conducted in which all raw marker positions were mathematically moved independently with the known Vicon position error with a distance and in a random direction in each frame, repeated a hundred times. Each time, the tibiofemoral movements were calculated.

**Results:**

The first experiment yields that the standard deviation of the calculated anterior tibia translation between marker setups was 0.88 mm and the standard deviation of the external tibia rotation between marker setups was 0.76 degrees. The second experiment yields that the standard deviation was 0.76 mm for anterior tibia translation and 0.38 degrees for external tibia rotation.

**Conclusion:**

A combined standard deviation of both experiments revealed that transients in anterior tibia translation less than 2.32 mm and external tibia rotations less than 1.70 degrees should be taken with caution. These results are 19.42% of the range of the anterior tibia translation and 13.51% of the rotation range during the jump task. The marker setup should be chosen carefully.

## Background

Hip centers and knee rotation axes can be calculated using a three-dimensional motion capture system. There are various methods to estimate the joint center of rotation and axis of rotation (i.e. [6, 7, 11, 14]). In this technical note the symmetrical center of rotation estimation (SCoRE) [8] and the symmetrical axes of rotation approach (SARA) [9] which are implemented in the software of the motion capture system Vicon (VICON Motion Systems Ltd, Oxford, UK) are investigated. Since the combination of these two methods can be used to calculate tibiofemoral movements [5] and are now easily available it makes sense to test its accuracy, which has not be done before. Being able to calculate these movements in demanding in vivo tasks may be of high interest in anterior cruciate ligament injury and reconstruction research. Currently in ACL research passive tibiofemoral movements are highly investigated, for example to compare surgical technics [1, 3] or to compare the results of non-copers with copers [4, 22]. However, it is found in literature that there is no correlation between passive and active anterior tibia translation (ATT) [12, 23]. To be able to evaluate the functional highly significant dynamic movement, such a method is very useful.

**Fig. 1:**
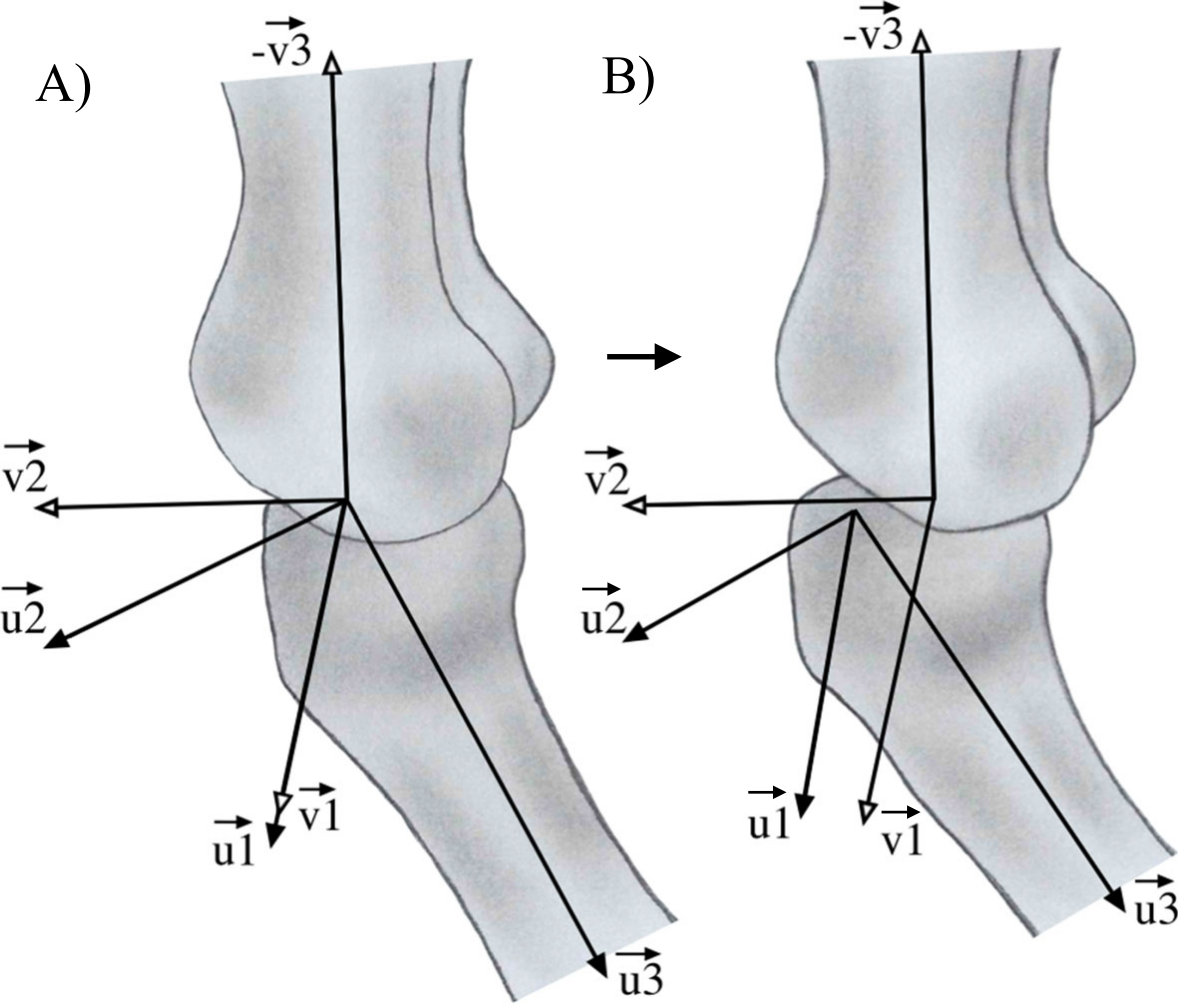
Coordination systems in the femur and tibia. Both coordination systems of the tibia and femur based on the SCoRE and SARA data. u1: the axis of rotation fixed in the tibia system; v1: the axis of rotation fixed in the femur system; u2 and v2: the cross product of the first and third axes; v3: vector from the center of rotation of the femur to the center of rotation of the hip; u3: vector from the center of rotation of the tibia to the center of the ankle. **a** Coinciding axis of rotation and therefore no anterior tibia translation. **b** Anterior translation and external rotation of the tibia

To calculate tibiofemoral movements, first the optimal common shape technique (OCST) should be performed [24]. Using the OCST, the markers on each segment are virtually replaced so that the markers of each segment act as a rigid body: the mutual distances between the markers do not chance over time. Based on the OCST markers, two coinciding points of rotation can be reconstructed in the knee using the SARA method. These points are estimated using dynamic calibration frames of a knee flexion-extension movement. One of these coinciding points is fixed in the rigid body of the tibia segment (*SARAtib*) and one in the rigid body of the femur segment (*SARAfem*) (Fig. 1a). In addition, based on the OCST markers, two centers of the knee (knee joint centers), one fixed in the rigid body of the tibia (*SCoREtib*) and one fixed in the rigid body of the femur (*SCoREtib*), can be calculated by the SCoRE method. Using these SARA and SCoRE data, two axes of rotation can be reconstructed in the knee, one in the tibia segment (*AXtib*) and one in the femur segment (*AXfem*). Moreover, based on the OCST markers, the point of rotation in the hip (hip joint center) can be calculated by the SCoRE method using dynamic calibration frames of a star-arc movement. For the mathematical model of these methods, see Ehrig et al. [8] and Ehrig et al. [9]. The dynamic translation and rotation of *AXtib* relative to *AXfem* estimate the tibiofemoral movements (Fig. 1b).

The reliability of the SCoRE and SARA methods including their application by Boeth et al. [5] have been studied several times [7, 16, 25, 26]. A high reliability (Intraclass correlation coefficient > 0.8) and no significant differences between five different observers who placed the marker set and between measure days was found in functional femur and tibia length (distances between the centers of the axis of rotation and the hip center or ankle center) calculated based on the SCoRE and SARA methods [25]. It was also reported that SARA showed a better inter-trial consistency of locations of the axis of rotation; however, worse consistency of the orientations of the axis of rotation compared to geometry-based axes while performing isokinetic knee flexion-extension [26]. Differences in external tibia rotation (ETR) relative to the femur between using SARA and fluoroscopic (invasive) techniques were reported between 5.7 and 9.6 degrees [16]. A correction equation led to a sum of the root mean square error of between 0.6 and 0.8 degrees for the SARA method [16]. In addition, De Rosario et al. [7] presented a mathematical model of soft tissue artefacts propagation to the position and direction of variable and fixed axes as calculated by three methods. One of these methods was the SARA method. They reported that SARA, measured in one subject, showed an absolute difference in position error of the measured and estimated axes of rotation of 6.3 mm and no method was superior to another. However, their marker setup was atypical: markers were placed relatively close to the knee.

A few studies have been published using the SCoRE and/or SARA method for research [5, 11, 14, 17, 20]. In the these studies, systematic errors could have been introduced. No studies are published on the influences of markers placements and measuring errors of the motion capture system on calculated tibiofemoral movements. The aims of the experiments in this technical note were to:

1. determine the sensitivity of the calculated tibiofemoral movements for the choice of the placement of the set of femoral markers.
2. determine the sensitivity of the calculated tibiofemoral movements for the errors in the captured positions of the Vicon markers found in previous research.

## Method

### Data

Tibiofemoral kinematics during a single leg hop for distance of one healthy subject (woman, 23 years old) were calculated using the SCoRE and SARA methods implemented in Nexus 2 of a Vicon system (10-camera, VICON MX-F40; VICON Motion Systems Ltd, Oxford, UK) in a lab of 5 by 10 meter and 3 meter high. The single leg hop for distance is used as the impact on the knee is greater than in common motion analysis experiments (gait, stair-ascending) and consequentially the sudden translations and rotations are believed to be greater. The sample frequency was 100Hz for each of the markers.

**Fig. 2:**
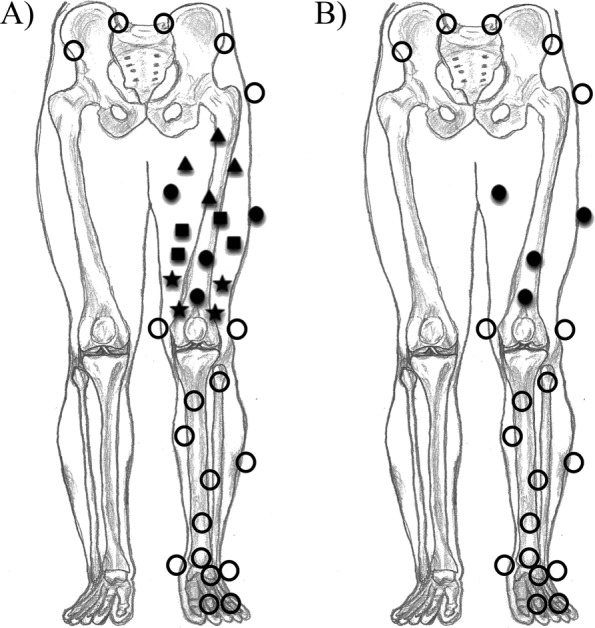
Marker setup. Marker setup of experiment 1 and 2. For both experiments, markers were attached on the right and left anterior and posterior superior iliac spine, greater trochanter, medial and lateral epicondyle of the femur, medial and lateral malleoli of the ankle; the heel; anterior on the proximal foot; and the first and fifth metatarsophalangeal joints. In addition, six additional markers were attached to the tibia. **a**) For experiment 1, sixteen additional markers were attached to the femur (four groups of four additional markers; the black filled markers with different shapes). **b**) For experiment 2, four additional femur markers were attached (filled black markers)

To be able to estimate the center of the hip, calibration frames of a star-arc movement of the tested leg were captured. To be able to estimate the coinciding rotation axes in the knee, calibration frames of an open kinetic flexion-extension movement with the tested leg raised from the ground was captured. Then, the subject performed six hops for distance with her preferred leg which was the left one. Markers were placed by the first author as shown in Fig. 2, adapted from Boeth et al. [5]. The motion capture of the hops were subsequently used to calculate the tibiofemoral movements with the method described in the next section.

### Computations of tibiofemoral movements

In order to compute tibiofemoral movements, based on the method of Boeth et al. [5], first OCST markers of all raw markers were calculated. Then, two coordinate systems were set up using a customized MATLAB (version 9.5, The MathWorks Inc., Natick, Massachusetts) script: one coordinate system in the femoral segment (parent system) and one in the tibia segment (child system). The first axes (*u*_1_ and *v*_1_) of both coordinate systems were calculated from the normalized axis of rotation of the corresponding segment calculated by the SCoRE and SARA methods (Fig. 1). The centers of these axes (centers of rotation; *u*_1*c*_ and *v*_1*c*_) were calculated by projecting the mean OCST corrected medial and lateral epicondyle markers on the rotation axes and were defined as the origin of the coordinate systems. The third axis of the tibia segment (*u*_3_) was the normalised vector from the center of rotation of the tibia (*u*_1*c*_) to the mean OCST corrected malleoli markers. The direction of this axis was changed to the opposite direction to make the system righthanded. The third axis of the femur segment (*v*_3_) was the normalized vector from the center of rotation of the femur to the center of rotation of the hip. The second axis of both coordinate systems (*u*_2_ and *v*_2_) were reconstructed by taking the cross product of the first and third axes of the coordinate systems. Hereafter, the coordinate systems were made orthogonal by another cross product of the first and second axes. In this way the direction of the first axis remains unchanged.

The coordinate systems U and V were defined from the collection of unit vectors *u*_1_,*u*_2_,*u*_3_ and *v*_1_,*v*_2_,*v*_3_ in their columns. The transpose of V and U were the rotation matrices of tibia and femur system (see (Additional file 1: Equation 1) and (Additional file 1: Equation 2) of the supplementary material). The rotation matrix from the tibia system to the femur system can be calculated from these two matrices ((Additional file 1: Equation 3) of the supplementary material). The femoral translation relative to the tibia is expressed in (Additional file 1: Equation 4) of the supplementary material. The euler rotation angles are given in (Additional file 1: Equation 5), (Additional file 1: Equation 6) and (Additional file 1: Equation 7) of the supplementary material, in which the indices represent the positions in the matrices (columns, rows). These euler angles are computed using the equations from Robertson [21]. The inverted femoral translation and the euler rotation angles relative to the tibia estimate the motion of the tibia relative to the femur.

### Experiment 1: the sensitivity of the measured tibiofemoral movements depending on the placement of the set of markers

On top of three bony landmark markers on the femur, sixteen additional markers were physically attached to the upper leg. For each single leg hop for distance, calculations of tibiofemoral movements were performed four times using different groups of four additional femur markers on different heights of the femur: one group of proximal femur markers (the triangles in Fig. 2a), one group of markers in the middle of the femur (the squares in Fig. 2a), one group of distal femur markers (the stars in Fig. 2a), and one group of markers spread over the femur (the black filled circles in Fig. 2a). This to study the effect of soft tissue artefacts (wobbling masses) of the upper leg.

In addition, calculations were performed using all sixteen additional femur markers. The results of any set of four femur markers were compared to the results where all markers were used, as it is believed on the basis from observing soft tissue motion in high speed video footage that using more markers will average out more soft tissue artefacts. Moreover, there is no golden standard.

### Experiment 2: the sensitivity of the measured tibiofemoral movements depending on vicon’s marker position errors

For this experiment the additional group of markers spread over the femur (shown in Fig. 2b) was used. A standard deviation of the position error of Vicon of 1.84 mm based on the error reported by Merriaux *et al* (2017) [15] with correction for our camera distance was used. The following two steps were performed 100 times for the same single leg hop:

1. All markers (raw data) were mathematically moved. For this, in each frame all markers were randomly displaced in a random direction. A normal distribution with a standard deviation of 1.84 mm was used as the amount of displacement and direction for each marker.
2. Each time, after displacement of the markers tibiofemoral movements were calculated.

This procedure was repeated for each 6 single leg hops for distance.

### Data analysis

For both experiments, the standard deviation of ATT and ETR for each frame between the 4 marker setups or the 100 trails were the markers were moved was calculated. Then, the mean of this standard deviation over time and over the 6 single leg hops for distance was calculated.

Also, the maximal, minimal and range of ATT and ETR was calculated for each frame. Then, the mean, maximal and minimal values of the range were calculated. To determine the effect of both the Vicon’s position errors and marker setup combined on the tibiofemoral movements, a combined standard deviation was calculated. For this only one single leg hop was used. The standard deviations of both experiments were squared and added together. Then, the square root of this value was calculated, which accounts for a combination of two standard deviations which have an independent origin.

## Results and discussion

### Experiment 1: the sensitivity of the measured tibiofemoral movements depending on the placement of the set of markers

**Fig. 3:**
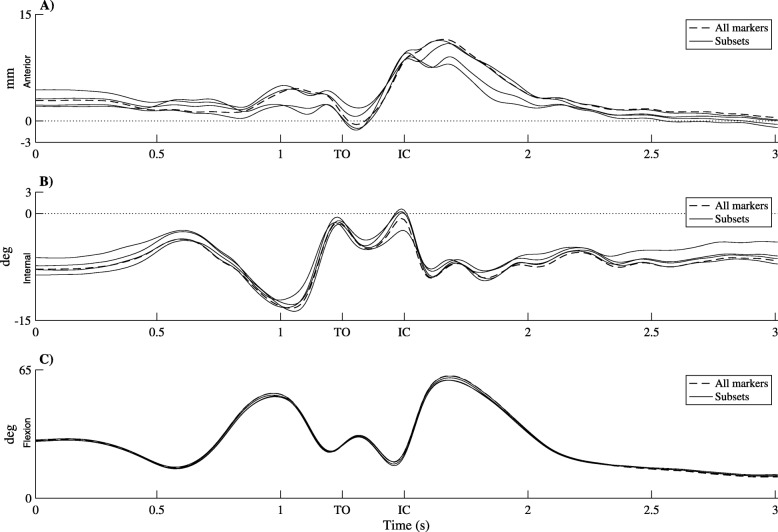
Results experiment 1. Tibia translation, tibia rotation and knee flexion angle of six single leg hops for distance over time with the different lines being computations with four different groups of femoral markers. The black dotted line is the situation where all sixteen femur markers where used. TO: too off; IC: initial contact. **a** Tibia translation. **b** Tibia rotation. **c** Knee flexion angle

The tibiofemoral movements resulting from the different marker groups have been depicted in Fig. 3. For the range of the ATT and ETR between results obtained with the different marker sets see Table 1. The standard deviation of the calculated ATT, when using different marker setups, is 0.88 mm and for ETR 0.76 degrees. To compare this result with the measurements: for the condition where all markers were used, the range of the ATT over the whole hop was 11.97 mm and of the ETR 12.58 degrees. When different marker setups are used, calculated differences during transients in ATT of less than twice the standard deviation (1.76 mm) and ETR (1.52 degrees) should be taken with caution.

**Table 1:** The mean, maximal and minimal range in anterior tibia translation (ATT) and external tibia rotation (ETR) between the different conditions and the mean standard deviation over time

	Experiment 1		Experiment 2	
	ATT (mm)	ETR (deg)	ATT (mm)	ETR (deg)
Mean StD over time	0.88	0.76	0.76	0.38
Mean of the range	1.91	1.72	3.82	1.93
Max of the range	4.46	3.08	3.93	2.03
Min of the range	0.85	0.46	3.79	1.87

Experiment 1: the sensitivity for the marker placement. Experiment 2: the sensitivity for Vicon’s position errors

See Table 2 for the mean, maximal and minimal difference between the results of the different marker setups compared with the results of the marker setup where all markers where used. The tibiofemoral movements computed with the marker setup where the markers were spread over the femur (the black filled circles in Fig. 2a) is most similar to the situation where all markers where used. This may be due to the cancelation of movements of soft tissue relative to the bone when applying markers over the whole femur, while as when using markers for example only proximal and medial on the femur, soft tissue artefacts may result in greater errors because these markers tend to move more as one group.

**Table 2:** Mean, minimal and maximal absolute differences between the results of the different marker setups compared with the results of the marker setup where all markers where used

	S1		S2		S3		S4	
	ATT	ETR	ATT	ETR	ATT	ETR	ATT	ETR
Mean difference	1.09	1.38	1.31	0.34	0.9	0.43	0.35	0.71
Min difference	0.00	0.01	0.00	0.00	0.00	0.00	0.00	0.13
Max difference	4.17	2.63	3.66	1.08	2.35	1.43	1.45	1.52

S1: proximal femur markers; S2: markers in the middle of the femur; S3: distal femur markers; S4: group of markers spread over the femur

These results imply that the marker setup should be chosen with care. We advise using a marker setup with markers spread over the femur (black filled circles in Fig. 2a) as in that case soft tissue artefacts may partly be canceled out.

### Experiment 2: the sensitivity of the measured tibiofemoral movements depending on vicon’s marker position errors

**Fig. 4:**
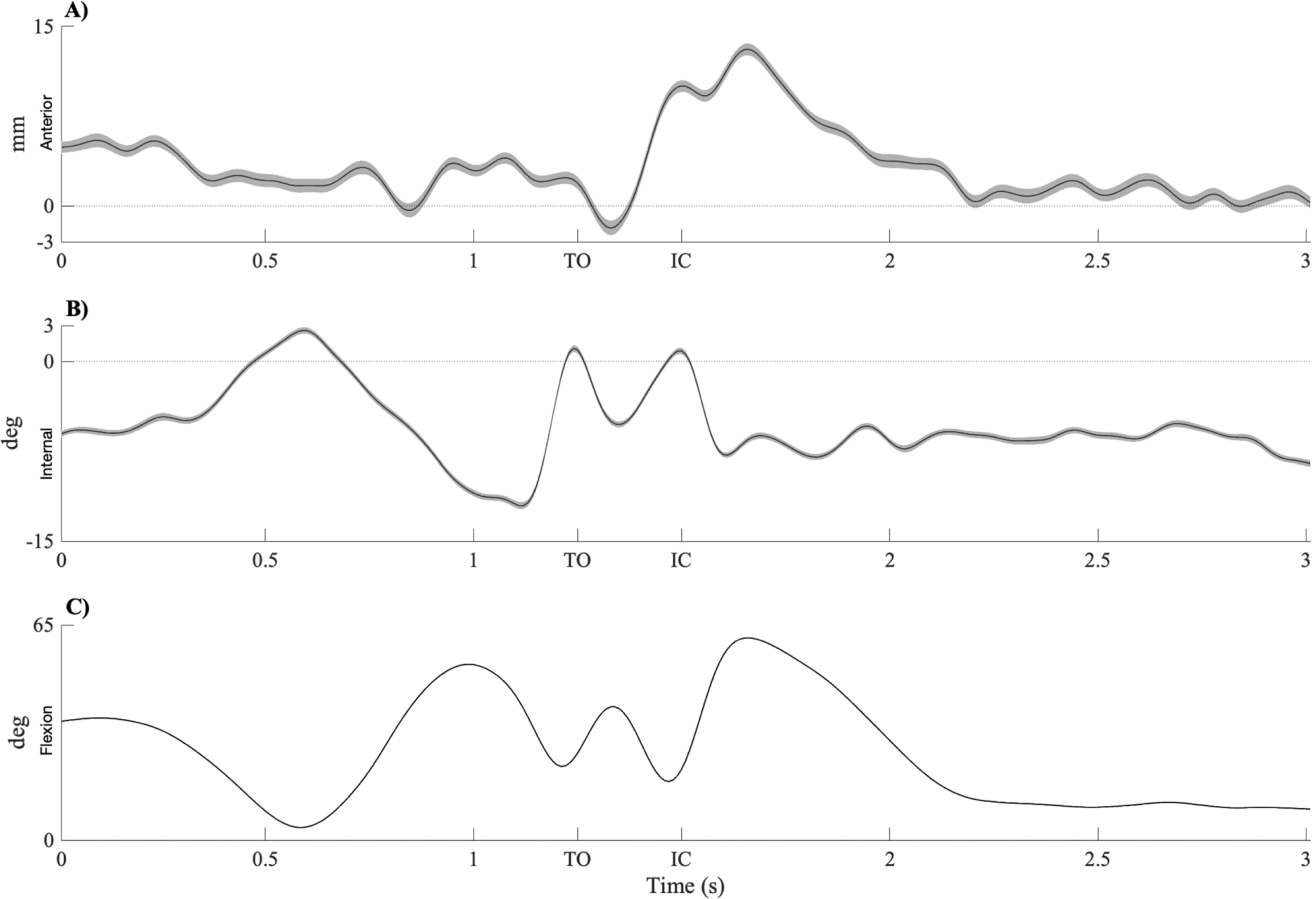
Results experiment 2. Tibia translation, tibia rotation and knee flexion of one single leg hop for distance over time. The black line is the mean of all trails. The grey area is the standard deviations in tibia translation and rotation of the 100 trials when all marker, per trail, were moved in a random direction. TO: too off; IC: initial contact. **a** Tibia translation. **b** Tibia rotation. **c** Knee angle

The mean and standard deviation of the tibiofemoral movements and the knee flexion angle resulting from all 100 trials where markers were moved have been depicted in Fig. 4. For the range of the tibiofemoral movements between the different trials see Table 1. The standard deviation between trials of the computed ATT and ETR was 0.76 mm and 0.38 degrees respectively.

These results imply that the position error of Vicon results in an error of ATT of 1.5 mm and ETR of 0.76 degrees (twice the standard deviation).

### The sensitivity of the measured tibiofemoral movements depending on both the vicon’s marker position errors and the placement of the set of markers

To determine the combined effect of both the Vicon’s position error and marker setup on the tibiofemoral movements, a combined standard deviation was calculated for one single leg hop for distance. For ATT this standard deviation was 1.16 mm and for ETR 0.85 degrees. The combination of the effect of the marker setup (wobbling masses) and Vicon’s position error may result in an error in tibiofemoral movements of twice the standard deviation; which is for ATT 2.32 mm and for ETR 1.70 degrees. These results are 19.42% of the range of the anterior tibia translation and 13.51% of the rotation range.

### Strong points and limitations

This is the first study which addresses the effects of the marker placement and Vicon’s position error on the ATT and ETR in a high demanding dynamic situation. The effects of marker placements and Vicon’s position error have been investigated [18, 19, 27]; however, as far as known to the authors no studies are published investigating those effects on tibiofemoral movements. A number of publications using the SCoRE and/or SARA method are relevant in light of such an investigation. The methods in the present sensitivity analysis and the ability to measure the ATT and ETR using such methods may be of high intrest in anterior cruciate ligament injury and reconstruction research, especially since the SCoRE and SARA methods are easily available.

Some possible limitations of this study need to be addressed. One possible limitation is that only one subject has been studied. The aim of this study was to observe the sensitivity of using different marker setups and the effect of Vicon’s position errors on the outcome measures of interest. For this, only one subject could be used on which the necessary parameters could be varied. We have chosen a subject who is active in recreational sport activities with a BMI of 22.7. This choice was made since the subject is representative of the population in which an ACL injury occurs frequently [10]. An addition to this study may have been to study subjects with very low and very high BMI in terms of wobbling masses. A future study could investigate this.

A second limitation may be the relative low frequency of 100Hz in which the marker positions were captured. This may have introduced loss of interesting data. However, 100Hz is ample to capture the frequency content of human movements, even during collisions such as with the ground.

Another possible limitation is that no data of a golden standard to measure the ATT and ETR is available, like bi-planar fluoroscopy data. Previous studies found an absolute range of ATT using bi-planar fluoroscopy model-based data during running of +/- 10 mm [2] and +/- 25 mm [13]. The results of Anderst et al. [2] are comparable to our results (12 mm). However, bi-planar fluoroscopy itself has its limitations [13]. A lack of a golden standard makes it impossible to verify the outcomes of the methods developed by Boeth et al. [5]. However, given the method, the present study seeks to find the effects of marker placement and measurement errors on the produced outcome measures. The results of the current study gives interesting information on determining ATT and ETR using the VICON motion capture system in dynamic situations. Being able to measure the ATT and ETR in high demanding tasks is of high interest in knee ligament research, for example to compare results after two different types of anterior cruciate ligament reconstruction or the results after different rehabilitation programs.

## Conclusion

Especially when different sets of markers are used, calculated differences in anterior tibia translation, i.e. between subjects, and transients of anterior tibia translations of less than 2.32 mm and of external tibia rotations of less than 1.70 degrees should be taken with caution. These results are 19.42% of the range of the anterior tibia translation and 13.51% of the rotation range. When using the SCoRE and SARA methods, the marker setup should be chosen carefully. We advise a marker setup with markers spread over the whole femur.

## Supplementary information

**Additional file 1** Equations

## Data Availability

The datasets used and/or analysed during the current study are available from the corresponding author on reasonable request.

## Abbreviations

ATT: Anterior tibia translation
ETR: External tibia rotation
SCoRE: Symmetrical center of rotation estimation
SARA: Symmetrical axes of rotation approach
OCST: Optimal common shape technique
