# Supplementary material for: Technical note: sensitivity analysis of the SCoRE and SARA methods for determining rotational axes during tibiofemoral movements using optical motion capture
Source: J Exp Orthop. 2020 Feb 10;7:6. doi: 10.1186/s40634-020-0219-z (PMC7010897; doi:10.1186/s40634-020-0219-z)
Supplement: Supplementary file 1 — Additional file 1 Equations [file 40634_2020_219_MOESM1_ESM.pdf]

Supplementary material: equations

$$M_{Tib} = U^T \tag{1}$$

$$M_{Fem} = V^T \tag{2}$$

$$M_{TibFem} = M_{Tib} * M_{Fem}^T \tag{3}$$

$$Tr = M_{Tib} * (u_{1c} - v_{1c}) \tag{4}$$

$$\alpha = \text{arctan2}\left(-M_{TibFem(3,2)}, M_{TibFem(3,3)}\right) \tag{5}$$

$$\beta = \arctan\left(\frac{M_{TibFem(3,1)}}{\sqrt{{M_{TibFem(1,1)}}^2 + {M_{TibFem(2,1)}}^2}}\right) \tag{6}$$

$$\gamma = \arctan\left(\frac{-M_{TibFem(2,1)}}{M_{TibFem(1,1)}}\right) \tag{7}$$
